# C5 Nerve Root Palsy: An Uncommon Postoperative Complication and Its Management

**DOI:** 10.7759/cureus.28988

**Published:** 2022-09-09

**Authors:** Nikita H Seth, Medhavi V Joshi, Pratik Phansopkar

**Affiliations:** 1 Musculoskeletal Physiotherapy, Ravi Nair Physiotherapy College, Datta Meghe Institute of Medical Sciences, Wardha, IND

**Keywords:** anterior decompression, case report, rehabilitation, c5 nerve root palsy, claudication, cervical fusion

## Abstract

The standard treatment for multiple levels of cervical prolapsed intervertebral disk (PIVD) is anterior cervical decompression and fusion. Although it is associated with positive outcomes, it is also fraught with complications. In this report, we present an unusual postoperative case of a 58-year-old male who underwent anterior decompression and cervical fusion at two levels - C4-C5 and C5-C6 - for traumatic PIVD and developed a postoperative complication of unilateral C5 motor palsy, making it difficult for the patient to elevate the shoulder. This postoperative complication had no known cause, but it could be iatrogenic or due to structural variation. There is sparse research on possible ways to avoid this complication. Physiotherapy management is critical in improving the patient's functional recovery. The neck and upper extremity functional measure scale and Neurogenic Claudication Outcome Score (NCOS) scale were used for measuring outcomes. The patient also had a two-year history of tingling and numbness in both lower extremities, which was treated conservatively. The difficulties that physiotherapists encounter in managing this uncommon postoperative complication in addition to the lumbar PIVD make it unique. The key to a better prognosis is early detection and management.

## Introduction

Anterior decompression and cervical fusion is performed to remove the herniated disc, relieve patients' symptoms, and ensure functional recovery [1]. The anterior approach for incision provides the benefit of easy accessibility to the disc without disturbing the spinal cord, nerves, and neck muscles [2]. Depending on the levels affected, the disc is removed and the space between the two vertebras is filled with a spacer to provide stability to the spine and achieve fusion. Although the procedure can lead to various postoperative complications, it is widely used to ensure better postoperative recovery. The various postoperative complications associated with the method include dysphasia, damage to recurrent laryngeal nerve, neck pain, and other rare complications including fifth cervical (C5) nerve root palsy, and adhesive capsulitis.

C5 nerve root palsy is a rare complication of anterior decompression and cervical fusion, occurring without any known etiology but could be possibly iatrogenic and caused by structural variations [3]. In most cases, there is pre-existing asymptomatic damage to anterior horn cells leading to severe postoperative C5 palsy. Hence, it is important to be alert to possible postoperative complications so that prompt and early identification and management can be ensured [4,5].

We present a patient who underwent anterior decompression and cervical fusion at multiple levels - C4-C5 and C5-C6 - for traumatic prolapsed intervertebral disc (PIVD) and then developed a postoperative complication of unilateral C5 nerve root palsy.

## Case presentation

Patient information

The patient was a 58-year-old male who presented to the hospital with the primary concerns of tingling in both upper and lower limbs along with pain in the lower back region. He also reported reduced use of his right upper extremity for the last two months due to the above complaints. The patient had a history of heavy weight lifting two months back following which he started experiencing the symptoms. Intermittent tingling in the right lower extremity more than the left was also present for the last 20 years, for which he was conservatively managed by using a lumbosacral belt. Pain in the lower back was described as radiating to the posterior aspect of both lower limbs. The pain was mild in severity, 5/10 on the numerical pain rating scale (NPRS), and dull aching in nature. The pain aggravated on walking, with a history of claudication present at 200 meters, and relieved on sitting. The patient did not have any other comorbidity.

Clinical findings

On physical examination, typical findings of multiple nerve lesions due to compression by a herniated disc in the cervical spine were seen (Table 1). A few special tests were found to be positive, including the straight leg raise (SLR) at less than 40 degrees suggesting lumbar disc disease, and the foraminal compression test suggestive of cervical disc disease. On postoperative day one, the strength of all upper limb muscles was noted to be grade 5/5 on the left side on Medical Research Council Grading System (MRCGS) and, on the right side, the strength of the deltoid and bicep was grade 4/5 with no sensory deficit. On preoperative examination, the strength of the upper limb muscle was noted to be grade 5/5 on the left side and, on the right, the strength of the deltoid and bicep was 5/5.

**Table 1:** Positive clinical findings of the cervical and lumbar assessment Motor and sensory loss at various dermatomal and myotomal levels was assessed

Nerve root	Positive findings	Present or absent
C5 nerve	Neck, shoulder, and scapula pain; lateral arm weakness during shoulder abduction, external rotation, elbow flexion, and forearm supination. The reflexes affected are the biceps and brachioradialis	Present
C6 nerve	Neck, shoulder, and scapula along with lateral arm, forearm, and hand pain. Weakness in shoulder abduction, external rotation, elbow flexion, and supination. Reflexes affected are the bicep and brachioradialis	Present
C7 nerve	Neck, shoulder, and middle finger pain are common, along with index and middle finger numbness. Weakness during radial extension and forearm pronation and wrist flexion. The reflex affected is the triceps	Present
S1 nerve	Back pain radiating into buttock, lateral or plantar foot. Sensory loss on the posterior calf, weakness on hip extension, knee flexion, and plantar flexion of the foot	Present but without bladder involvement

Timeline of the current episode

The patient was admitted on 6/12/2021 to the hospital and, after a complete examination, a diagnosis was made of cervical disc disease with PIVD at C3-C4, C4-C5, and C5-C6 with lumbar disc disease with PIVD at L5-S1 with neuro-deficit. After mandatory screening for coronavirus disease 2019 (COVID-19), hepatitis B surface antigen (HBsAg), hepatitis C, and HIV, the patient was scheduled to undergo anterior decompression and spinal fusion at C4-C5 and C5-C6 on 8/12/21. The patient was then shifted to the recovery room for a day and then to the general ward for further care and conservative management of lumbar PIVD. The patient was then prescribed a home exercise program.

Diagnostic assessment

**Figure 1:**
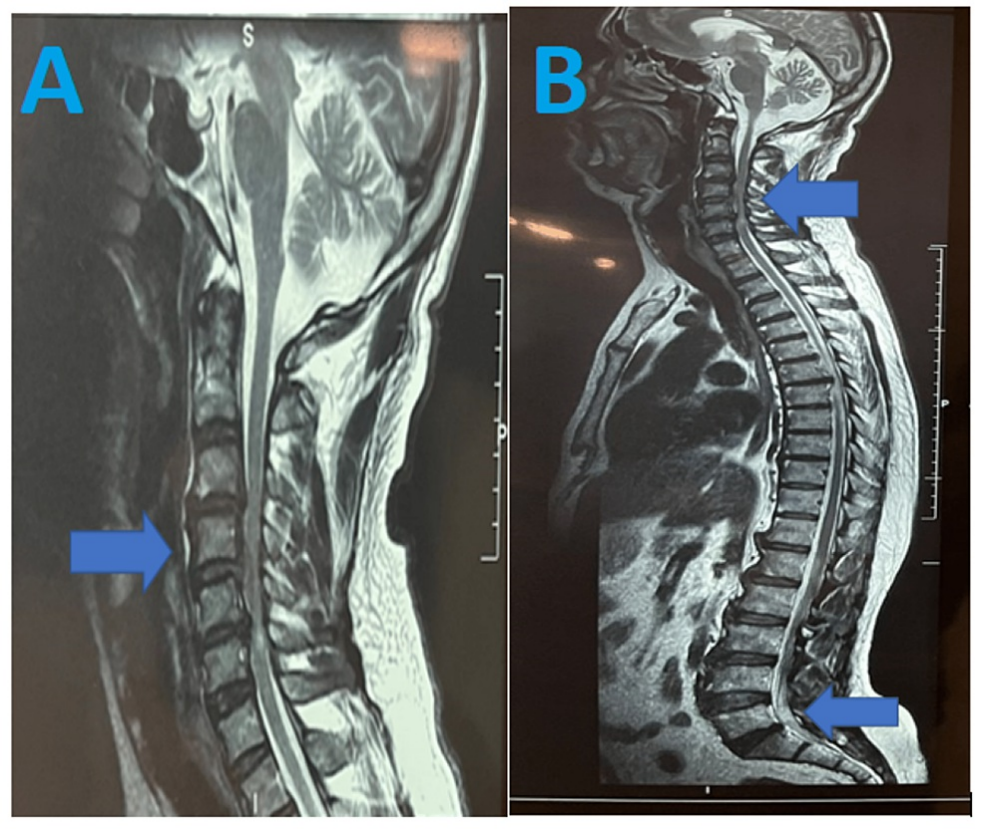
MRI of the cervical spine (A) The blue arrow indicates multiple levels (C3-C6) where diffuse disc bulge indenting over the thecal sac is seen. (B) MRI of the full length of the spine. The upper blue arrow shows a disc bulge at the cervical spine and the lower blue arrow is indicative of a disc bulge between the fifth lumbar and first sacral vertebrae MRI: magnetic resonance imaging

**Figure 2:**
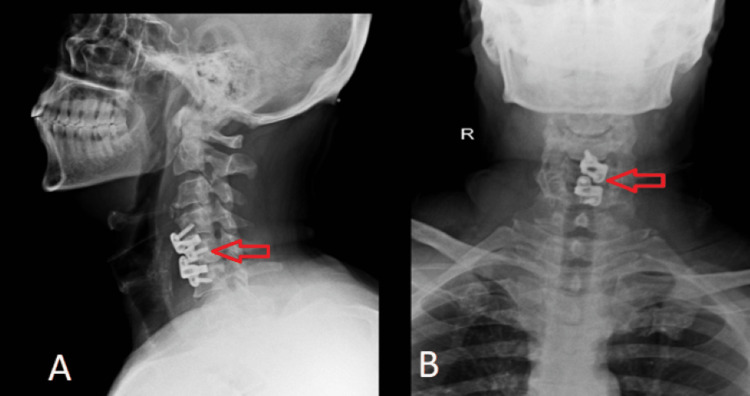
Postoperative X-ray of the cervical spine (A) Lateral view of X-ray where arrows indicate the placement of Jayon spacer between C4-C5 and C5-C6 level. (B) Anteroposterior view of the X-ray; red arrows indicate the spacer at multiple levels

An MRI scan plays a very important role in diagnosing the exact location of the lesion. The MRI scan of the cervical spine in our case revealed marginal anterior and posterior osteophytes at multiple cervical levels. At the C3-C4 disc level, a diffuse disc bulge intending over the anterior thecal sac causing narrowing of bilateral neural foramina was noted, along with a posterior annular tear at that level. At the C4-C5 disc level along the disc bulge, there was posterior-central disc protrusion causing narrowing of bilateral foramina. There was an associated posterior annular tear and spinal canal stenosis (4 mm) at this level. At the C5-C6 level, there was a diffuse disc bulge intending over the anterior thecal sac with left posterolateral disc protrusion with spinal canal stenosis (3 mm) at this level, and a disc bulge was seen at the L5-S1 disc level (Figures 1-2). In light of these findings, anterior decompression and cervical fusion were done in which two spacers of 5 mm each were placed at C4-C5 and C5-C6 regions. Then, a postoperative X-ray was taken for confirming the position of the spacer.

Diagnosis

On the basis of MRI and clinical presentation, the patient was diagnosed with cervical disc herniation with radiculopathy, for which he was planned to be managed surgically.

Therapeutic interventions

Surgical Intervention

**Figure 3:**
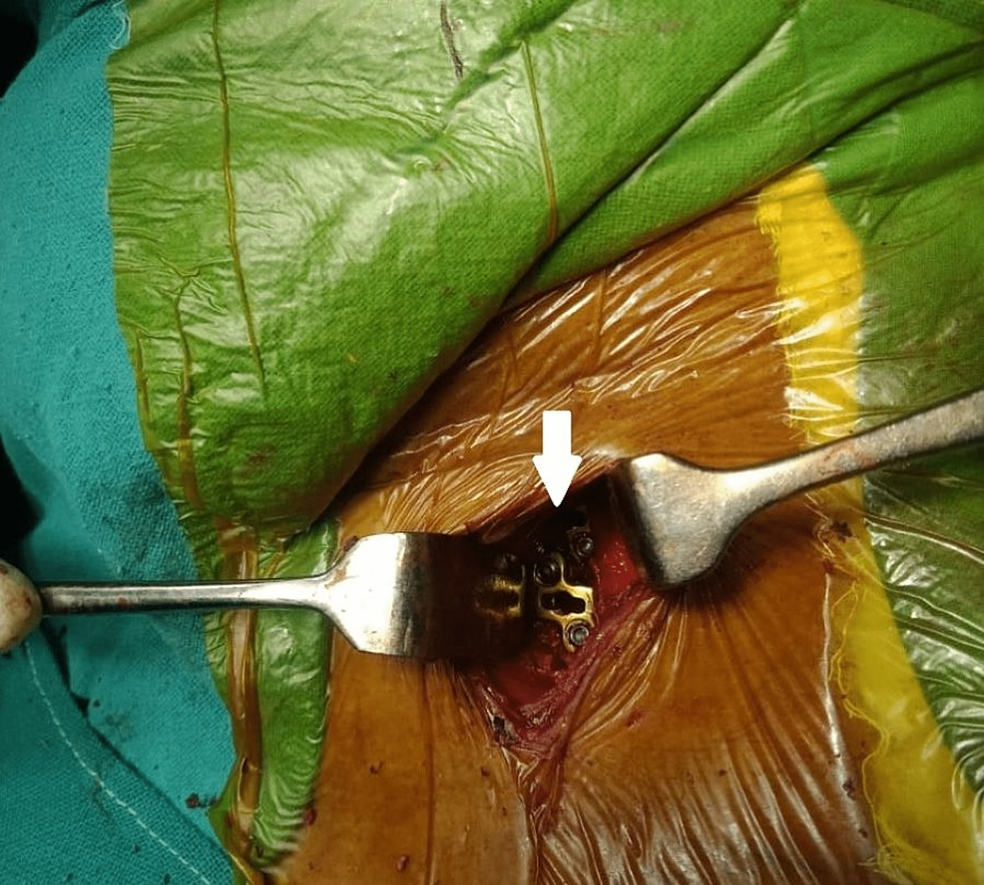
Intraoperative image The white arrow shows a 2-mm Jayon spacer

The patient underwent anterior decompression cervical spine fusion surgery, which is a standard and widely accepted treatment of choice for severe cervical PIVD patients as it provides the benefit of easy accessibility to the disc without causing much damage to the nearby structures while also providing the stability to the spine. The patient was given general anesthesia in the supine position and a longitudinal incision of around 5 cm was done over the left sternocleidomastoid muscle. Osteophytes at the end plate at the C5 vertebra were removed. Discectomy at C4-C5 and C5-C6 was done. Two Jayon spacers of 2 mm were fixed with four screws (Figure 3). The mini drain was then placed.

Physical Therapy Rehabilitation

**Figure 4:**
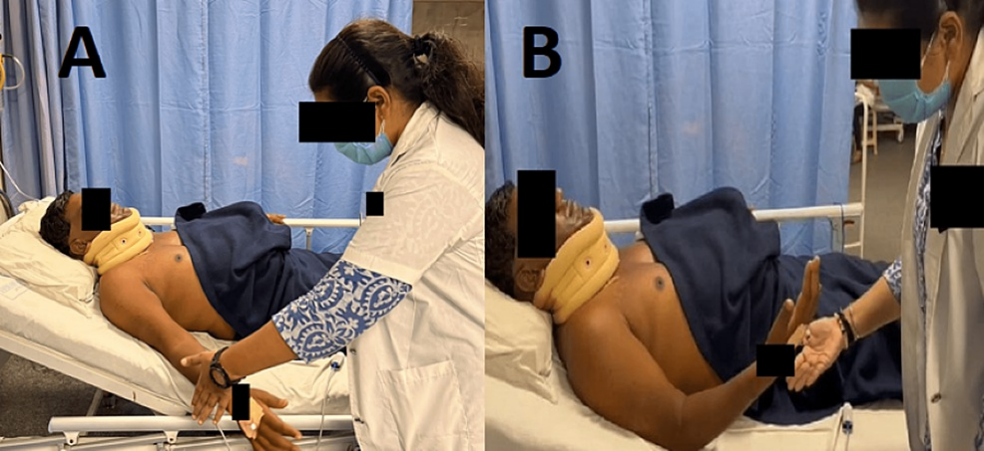
Rehabilitation exercises for the upper limb (A) Shoulder abduction and (B) elbow flexion with minimal guidance by the therapist's hand in the desired direction of motion

The intervention was started when the patient was shifted to the recovery room after eight hours of surgery. On physiotherapy assessment, the patient was diagnosed with C5 motor palsy. Hence, along with postoperative management for the cervical spine (Table 2), the patient was additionally managed for C5 nerve root palsy (Table 3) (Figure 4) and lumbar spine PIVD (Table 4).

**Table 2:** Postoperative physiotherapy care for cervical spine

Serial no.	Goal	Intervention	Rationale
1	Patient education	(A) Educating the patient about the importance of physiotherapy for improving his condition and gaining functional recovery. (B) Precautions that are to be taken for a few weeks to prevent secondary postoperative complications: 1. Avoid driving for at least four weeks. 2. Avoid heavy weight lifting	It helps to improve treatment effectiveness and increases the patient’s dedication to the treatment
2	To maintain the strength of neck muscles	Neck isometric exercises involving five repetitions: one set of each exercise progressing to 10 repetitions	Isometric contraction helps in the inhibition of pain and stimulates the healing process
3	To improve swallowing ability post-surgery	Shaker exercise and Masako maneuver were explained to the patient and he was advised to perform them thrice a day, beginning with 10 repetitions throughout the day and progressing if the pain does not persist	(1) Helps in improving strength and ability to swallow. (2) Improves the function of pharynx constriction by strengthening the muscle. (3) Helps in improving coordination of the larynx and hyoid bone
4	To reduce anxiety and improve breathing capacity	Diaphragmatic breathing exercise: twice a day with 30 repetitions throughout the day	Encourages full oxygen exchange and improves blood flow

**Table 3:** Physiotherapy rehabilitation for postoperative C5 motor palsy PNF: proprioceptive neuromuscular facilitation

Serial no.	Goal	Intervention	Rationale
1	To reduce pain and facilitate shoulder movements	Postoperative day three to week two: Pendulum or Codman`s exercise (to and fro in flexion and extension and in a circular motion), five repetitions twice a day	Provides mild joint distraction and reduces pain
2	To improve the strength of the deltoid and bicep muscles	Postoperative day three to week one: isometric shoulder abduction exercises (10 repetitions with two sets); isometric elbow flexion exercises (10 repetitions with two sets). Week two to week four: active resisted exercises using manual resistance along with PNF, a technique that involves dynamic reversals using the functional task.	Facilitates muscle firing to begin to re-establish neuromuscular control but protects healing tissues

**Table 4:** Physiotherapy rehabilitation for lumbar prolapse intervertebral disc disease

Serial no.	Goal	Intervention	Rationale
1	To reduce low back pain	Avoiding bending and lifting activities. Hot fermentation for 10 minutes twice a day	It helps to improve blood supply and helps to restore movements
2	To promote muscle relaxation	Gentle active pain-free range of motion exercises (10 repetitions each)	Maintains the length of muscle fibers and reduces tissue tension
3	To restore muscle strength	Week two to four: stretching and flexibility exercises; stretching of hamstrings, quadriceps, gluteal stretch, piriformis stretch, isometric hip adduction (three sets with 15-second holds for each)	Helps to reduce the tension on the neurological structure and reduces symptoms of claudication and tingling
4	To improve the strength of abdominal exercises	Core strengthening exercise: transverse abdominis activation during core strengthening exercises included: bridging (10 repetitions with five-second hold), dead bug exercise (10 repetitions with three sets)	Improves proprioception in the lumbar spine

Follow-up and outcome of interventions

**Figure 5:**
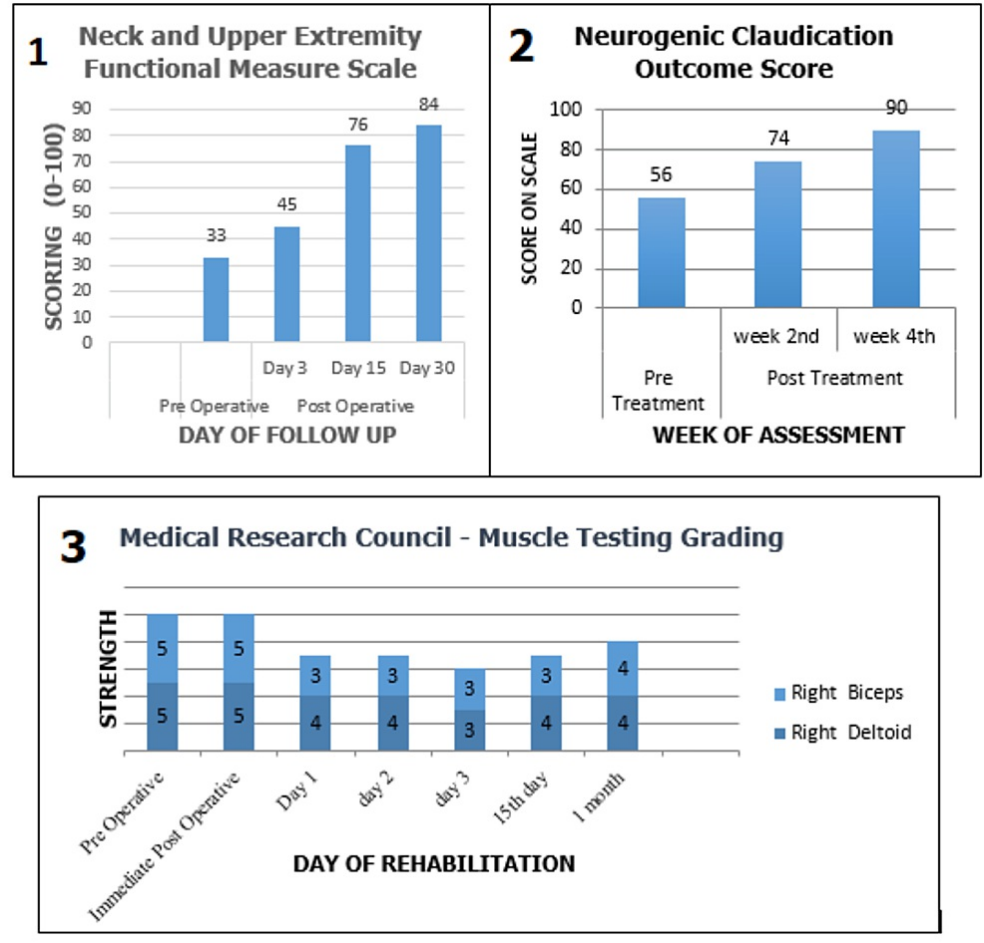
Graphical representation of outcome measures Graph 1: presents the comparison of the neck and upper extremity functional scale on preoperative day zero and postoperative days 3, 15, and 30. Graph 2: represents a comparison of the Neurogenic Claudication Outcome Score taken prior to the administration of the physical therapy treatment and values that were taken in the second and fourth weeks post-initiation of treatment. Graph 3: shows the initial decline and the gradual rise in the strength of the biceps and deltoid muscles post-rehabilitation taken at various intervals by manual muscle testing

The neck and upper extremity functional scale or neck disability index are used to assess the difficulty that individuals experience while performing activities of daily life, with a total score of 100 indicating no difficulty at all. The Neurogenic Claudication Outcome Score (NCOS) is an expansion of the Low Back Outcome Score developed by Greenough: a scale used to measure the severity of symptoms of claudication. It consists of eight questions which range from those related to when the symptoms begin, their severity, and to those that touch on the extent to which the symptoms are affecting the common activities of daily living. Each question has a choice of four answers from a to d with option ‘a’ being allotted 0 points and ‘d’ 6 points. A maximum score of 100 can be attained, which would indicate no asymptomatic condition. The patient had a post-rehabilitation score of 90, indicating minimal symptoms. The Medical Research Council muscle testing grading system is used to grade the strength of the muscle as assessed by manual muscle testing. It consists of 0-5 grades with 0 indicating no contraction and 5 indicative of the full range of motion against gravity, with maximal resistance. The comparison of preoperative and treatment values with postoperative values for the three outcome measures is represented graphically (Figure 5).

## Discussion

According to a 2012 study by Kim et al., the prevalence of C5 nerve root palsy after anterior decompression and cervical fusion at multiple levels is 4.3% [6]. There is some debate about the exact cause, but the most likely cause has been identified as nerve root injury or spinal cord shifting resulting from nerve traction [7,8]. Despite being a debilitating complication, it is frequently overlooked in patients. It could cause deltoid and bicep muscle weakness, as well as loss of sensation in the C5 dermatome [9]. Our patient complained of cervical PIVD with a history of heavy weight lifting, as well as symptoms of lumbar PIVD due to age-related degenerative changes in the lumbar spine. Physical therapy has been shown to be effective in improving deltoid and bicep strength following C5 nerve root palsy and in managing mild lumbar PIVD conservatively. Kim SW et al.'s 2017 study reported on the successful conservative management of bilateral C5 nerve root palsy in a 58-year-old female patient [10].

Physiotherapy played a critical role in this patient's recovery by preventing surgery, enabling the patient to be managed conservatively for lumbar PIVD, and treating the patient for C5 motor palsy. Physiotherapy rehabilitation improved the patient's upper extremity functional independence while also alleviating the neurologic symptoms of claudication caused by lumbar PIVD. The current study discovered that after one month of intervention, the strength of the deltoid and bicep muscles improved. As a result, physiotherapy has been shown to be extremely beneficial. This case is unique in its own right due to the multiple pathologies and multidisciplinary approach used in treating that helped with improving muscle strength, reducing symptoms, and improving overall functional independence in a patient with postoperative side effects of C5 palsy following anterior decompression and cervical fusion at two levels along with lumbar PIVD.

## Conclusions

We discussed the case of a patient with postoperative complications of C5 palsy following anterior decompression and cervical fusion at two levels, as well as lumbar PIVD. This report suggests that a multi-disciplinary approach, including medical, surgical, and physiotherapy rehabilitation, can prove to be highly beneficial in treating these patients in terms of improving muscle strength, reducing symptoms, and improving overall upper extremity functional measures. Early diagnosis and management of postoperative complication aid in achieving faster recovery and better prognosis.
